# The increased sensitivity of qPCR in comparison to Kato-Katz is required for the accurate assessment of the prevalence of soil-transmitted helminth infection in settings that have received multiple rounds of mass drug administration

**DOI:** 10.1186/s13071-020-04197-w

**Published:** 2020-06-24

**Authors:** Julia C. Dunn, Marina Papaiakovou, Kay Thwe Han, Darren Chooneea, Alison A. Bettis, Nay Yee Wyine, Aye Moe Moe Lwin, Nay Soe Maung, Raju Misra, D. T. J. Littlewood, Roy M. Anderson

**Affiliations:** 1 Department of Infectious Disease Epidemiology, School of Public Health, Faculty of Medicine, Imperial College London, London, W2 1PG UK; 2 London Centre for Neglected Tropical Disease Research, London, UK; 3 Department of Life Sciences, Natural History Museum, London, UK; 4 Department of Medical Research, Ministry of Health and Sports, Nyapyitaw, Myanmar; 5 Core Research Laboratories, Natural History Museum, London, UK; 6 University of Public Health, Myorma Kyaung Street, Yangon, 11131 Myanmar

**Keywords:** Soil-transmitted helminths, Kato-Katz, qPCR, Diagnostics, Monitoring and evaluation

## Abstract

**Background:**

The most commonly used diagnostic tool for soil-transmitted helminths (STH) is the Kato-Katz (KK) thick smear technique. However, numerous studies have suggested that the sensitivity of KK can be problematic, especially in low prevalence and low intensity settings. An emerging alternative is quantitative polymerase chain reaction (qPCR).

**Methods:**

In this study, both KK and qPCR were conducted on stool samples from 648 participants in an STH epidemiology study conducted in the delta region of Myanmar in June 2016.

**Results:**

Prevalence of any STH was 20.68% by KK and 45.06% by qPCR. Prevalence of each individual STH was also higher by qPCR than KK, the biggest difference was for hookworm with an approximately 4-fold increase between the two diagnostic techniques. Prevalence of *Ancylostoma ceylanicum*, a parasite predominately found in dogs, was 4.63%, indicating that there is the possibility of zoonotic transmission in the study setting. In individuals with moderate to high intensity infections there is evidence for a linear relationship between eggs per gram (EPG) of faeces, derived from KK, and DNA copy number, derived from qPCR which is particularly strong for *Ascaris lumbricoides*.

**Conclusions:**

The use of qPCR in low prevalence settings is important to accurately assess the epidemiological situation and plan control strategies for the ‘end game’. However, more work is required to accurately assess STH intensity from qPCR results and to reduce the cost of qPCR so that is widely accessible in STH endemic countries.
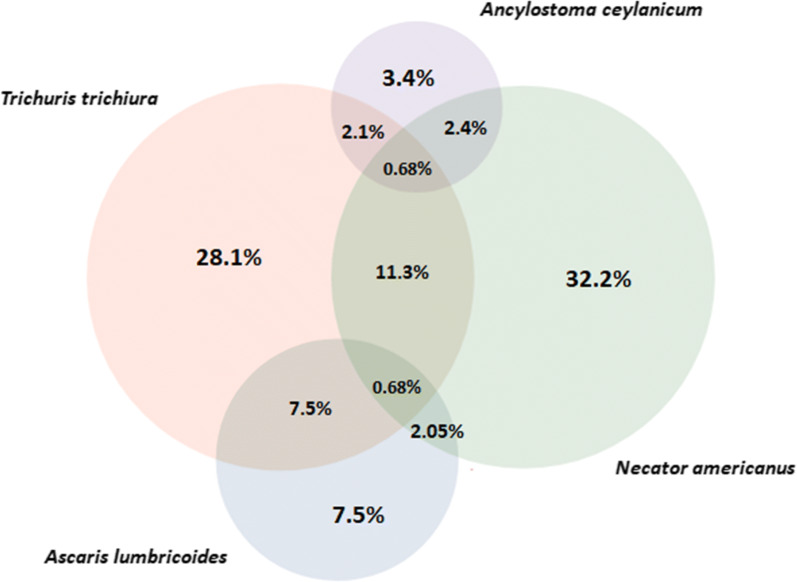

## Background

Infection with soil-transmitted helminths (STH; *Ascaris lumbricoides*, *Trichuris trichiura*, *Ancylostoma duodenale* and *Necator americanus*) affect approximately 1.4 billion people globally [1]. Mass drug administration (MDA) programmes have decreased overall STH prevalence and intensity of infection in many settings to the point that the interruption of transmission and parasite elimination are feasible goals in countries with consistently high MDA coverage over many rounds [2–4]. Analyses based on stochastic models of parasite transmission and controls have calculated that if the true prevalence is reduced below a threshold of 2%, there is an 80% positive predictive value (PPV) that transmission of STH has been interrupted and infection will not bounce back to endemicity, although this conclusion is sensitive to the baseline prevalence prior to the use of MDA (the magnitude of the basic reproductive number R_0_) [3, 5]. For accurate assessment, monitoring and evaluation (M&E), diagnostics with very high sensitivity are required to accurately measure a prevalence as low as 2% [6].

Currently, the diagnostic tool most frequently used to identify STH infection is the Kato-Katz (KK) technique [7]. The KK technique consists of examining sieved stool *via* microscopy and counting the number of eggs on the slide. The egg count is then multiplied by 24 to give eggs per gram of faeces (EPG). The World Health Organization (WHO) defines EPG thresholds to group infection intensity in a person into low, medium or high [8]. These vary between species as different species produce varying mean number of eggs per fertile female worm [9]. A further complication is density dependence, when female worms produce fewer eggs as the worm burden within an individual increases (i.e. there is more competition for resources within the host) [10, 11].

The KK technique is widely used because it is inexpensive, easy to prepare, requires little equipment and can be used in the field without reliable electricity [12, 13]. However, the sensitivity of KK decreases in low prevalence and low intensity settings [14–16]. Disadvantages of KK include the lower sensitivity of the diagnostic for hookworm, as hookworm eggs desiccate on the slide after approximately one hour, and the inability to visually distinguish between the hookworm species on the slide (*An. duodenale*, *N. americanus* and *Ancylostoma ceylanicum*) [17]. The requirement for a more sensitive diagnostic tool, as well as the interest in distinguishing whether an infection is with a STH species that is predominately found in humans or animals [18, 19], has led to increasing research into the use of the polymerase chain reaction (PCR) as a STH diagnostic [16, 20, 21].

Quantitative PCR (qPCR) is widely used in the biological sciences to detect and quantify target DNA in biological samples [22]. Previous studies have compared qPCR results to KK results for the same individuals and have found qPCR to be more sensitive, detecting more positive infections [16, 23]. This suggests that, in low STH prevalence and low intensity settings, epidemiology studies that solely use the KK technique may be missing a proportion of positive infections and therefore underestimating prevalence. Questions remain about the quantitative results of qPCR, usually reported as C_t_ values or DNA copy numbers, and whether they can be directly related to STH infection intensity and EPG counts [24]. Egg-spiking studies have attempted to directly correlate DNA copy number with one STH egg [16, 25] but issues regarding DNA loss through the DNA extraction and qPCR protocols limit the value of these studies. There has also been extensive work on optimising the qPCR protocol, testing different DNA extraction techniques and combinations of primers and probes [20, 21, 26]. A study by Easton et al. [27] found that the greatest source of variation in qPCR results was the identity of the stool donor (i.e. each individual’s actual STH infection intensity) and not technical factors related to DNA extraction procedure and subsequent plate preparation for PCR analysis [27]. Nevertheless, for qPCR to be validated as a gold-standard diagnostic for STH, more data needs to be gathered from different geographical areas and at different levels of STH endemicity [28, 29].

In this study we used qPCR to diagnose STH infection in stool samples collected from two villages in the delta region in Myanmar [30]. We compared individual qPCR results to KK results derived from the same stool sample. We also analysed the variability in cycle threshold (C_t_) values and DNA copy number when doing multiple DNA extractions on the same stool sample to assess how reliable a single qPCR is for accurately diagnosing infection and quantifying the intensity of infection.

## Methods

### Sample collection

Stool samples were collected during the final survey of an epidemiology study of STH infection in the delta region of Myanmar, conducted in June 2016. Methods for the study have been detailed in previous publications [31]. A single stool sample was collected from 665 participants enrolled in the study. Microscopy for KK measures was carried out in field laboratories set up in the study villages. Approximately 2 g of stool was placed in a tube (no preservative was used), labelled with the participant’s ID code and placed in a cooled container. Samples were transported to the University of Public Health, Yangon (UoPH) at the end of each day and stored in a − 20 °C freezer. Samples were shipped to Imperial College London on dry ice in March 2018 and onto the Natural History Museum, London, UK in June 2018 and stored in a − 80 °C freezer. Quantitative PCR was carried out at the Natural History Museum, London, UK.

### Kato-Katz

All stool samples were examined microscopically immediately on collection. Samples were prepared and examined following the standard Kato-Katz technique [7]. The numbers of *Ascaris lumbricoides*, *Trichuris trichiura* and hookworm eggs were counted and recorded. All slides were read for hookworm eggs within an hour of preparation, before desiccation of the hookworm eggs. Slides were then set aside to clear for at least a further hour to allow *A. lumbricoides* and *T. trichiura* eggs to become visible. One slide per participant was prepared and read. For quality control, a random 10% of slides were read by two laboratory technicians.

### DNA extraction

DNA was extracted from each stool sample using the MP Bio Fast DNA Spin kit for Soil (MP Biomedicals, Santa Ana, CA, USA). The modified DNA extraction protocol has been previously described [21] and includes an addition of internal control to validate the success and consistency of DNA extraction. Briefly, stool samples underwent a lysis step followed by bead beating to break open helminth eggs and release DNA. Following multiple steps of DNA purification from contaminants (organic matters, salts, inhibitors etc), the eluted DNA was dissolved in nuclease-free water. After extraction DNA samples were kept frozen at − 20 °C until testing by qPCR.

### qPCR

Prior to qPCR, DNA samples were slowly thawed by placing them in a 3 °C refrigerator for 30 min. The primer and probe sequences used have been previously published in [21] and [32]. Each plate was prepared and tested for a single species (*Ascaris lumbricoides*, *Trichuris trichiura*, *Necator americanus*, *Ancylostoma duodenale*, *Ancylostoma ceylanicum*) or for an internal amplification control (IAC). Reaction volumes of 7 µl were loaded onto a 96-well Fast MicroAmp plate (Applied Biosystems) consisting of 2 µl of DNA, 3.5 µl TaqPath ProAmp Master Mix and 1.5 µl of species-specific primers, probes and nuclease-free water. The plates were loaded with 39 DNA samples (in duplicate), three no template controls (NTC) (2 µl of nuclease-free H_2_O instead of DNA) and, for quantification, five serial dilutions of positive standards, consisting of double-stranded target molecules, commercially produced by qStandard (Edgware, UK) using DNA extracts identified as positive for that particular STH (run in triplicate, at 1:2 dilutions). Plates were run on a StepOne Plus real Time PCR System (Thermo Fisher Scientific, Loughborough, UK).

A cycle threshold (C_t_) was automatically calculated by the qPCR software for each plate based on the results from that plate. A negative result was determined by the sample exhibiting no amplification for the target species but exhibiting amplification for the internal control. In addition, any late amplifications after the cycle threshold were deemed negative. A positive result was determined by the sample exhibiting amplification before the cycle threshold and the internal control also exhibiting amplification. Whenever the two replicates run per sample were not in agreement (i.e. one replicate was positive, one replicate was negative), the qPCR for that sample was repeated. Any samples where the internal control was not detected underwent DNA extraction again and the qPCR was also repeated. The number of DNA copies in each sample was calculated from a standard curve defined by linear regression of the positive standard controls included in each plate. The qPCR efficiency was calculated by the slope of the curve and any plates with efficiency below 90% were re-run.

### Statistical analysis

Of the 665 samples that underwent qPCR, 648 had recorded KK results; all analyses in this paper use data from these 648 participants. As all samples were run in duplicate on the PCR plates, a mean C_t_ and DNA copy number was taken between the two wells. As 2 µl of sample DNA was loaded into each well, the mean DNA copy number was multiplied by 1.75 to correct for the dilution in each well (2 µl DNA in 7 µl solution) and to give copy number per µl. Exact confidence intervals (95% two-sided) for mean prevalence were calculated using the Clopper-Pearson method [33]. It is not possible to differentiate the hookworm species by the KK technique due to visual similarity of *N. americanus*, *An. duodenale* and *An. ceylanicum* eggs. Therefore, for analyses that compare KK and qPCR for hookworm, an aggregated hookworm result was calculated from the qPCR results. If an individual was infected with any one of the three hookworm species by qPCR they are counted as hookworm-infected. For analyses of intensity of infection, the DNA copy numbers/µl were summated over all hookworm species for a final hookworm DNA copy number. Non-parametric mean EPG adjusted percentiles (95% two-sided, bias-corrected and accelerated (BCa)) were calculated using bootstrapping methodology with the *boot* package. The WHO recommended intensity cut-offs were used to group individual EPG into low, medium and high intensity infections [8]. McNemar’s test was used to assess the differences in prevalence measured by KK and qPCR. The Kendall rank correlation test was used to analyse the correlation between DNA quantity and EPG. The Kendall Tau-b value was chosen as it adjusts for tied ranks. Kendall Tau-b values range between minus one (all pairs are discordant) and one (all pairs are concordant); a higher Tau-b value indicates more concordant than discordant pairs of individual egg counts and therefore higher overall correlation [34]. The Sign test was used to assess the difference between C_t_ values for the first and second DNA extraction. RStudio (R version 3.0.1, Vienna, Austria) was used for the following statistical analyses and to create the figures.

## Results

### Prevalence of STH by qPCR and comparison to Kato-Katz

The prevalence of infection with STH was found to be higher when using qPCR as the diagnostic tool than using KK. Of the 648 participants who provided stool samples and had recorded Kato-Katz results, prevalence of infection with any STH species was 45.06% (95% CI: 41.18–48.98%). The most prevalent STH was *T. trichiura* (22.84%), followed by *N. americanus* (22.69%), *A. lumbricoides* (8.80%), *An. ceylanicum* (4.63%) and *An. duodenale* (0.15%). Combining the three hookworm species, prevalence of hookworm was 25.62% (95% CI: 22.30–29.16%). Table 1 presents the prevalence results for Kato-Katz and qPCR as well as the results of significance tests to test the differences between the diagnostics. There was a statistically significant difference in prevalence as diagnosed by KK or qPCR for all species. There was a percentage increase (KK result as the denominator) of 128.00%, 80.49% and 295.37% for *A. lumbricoides*, *T. trichiura* and hookworm prevalence, respectively.

**Table 1:** Number of positive samples (n), prevalence (%) and 95% confidence intervals (95% CI) for Kato-Katz and qPCR diagnostic

Species	Kato-Katz		qPCR		*P-*value
	*n*	% (95% CI)	*n*	% (95% CI)	
Any STH	134	20.68 (17.62–24.00)	292	45.06 (41.18–48.98)	< 0.0001
*Ascaris lumbricoides*	25	3.89 (2.51–5.64)	57	8.80 (6.73–11.25)	< 0.0001
*Trichuris trichiura*	82	12.65 (10.19–15.46)	148	22.84 (19.66–26.27)	< 0.0001
Hookworms	42	6.48 (4.71–8.66)	166^a^	25.62 (22.30–29.16)^a^	< 0.0001^a^
*Necator americanus*			147	22.69 (19.51–26.11)	
*Ancylostoma duodenale*			1	0.15 (0.00–0.86)	
*Ancylostoma ceylanicum*			30	4.63 (3.15–6.54)	

^a^ Calculated from aggregated hookworm qPCR results

*Abbreviation*: STH, soil-transmitted helminth

**Fig. 1:**
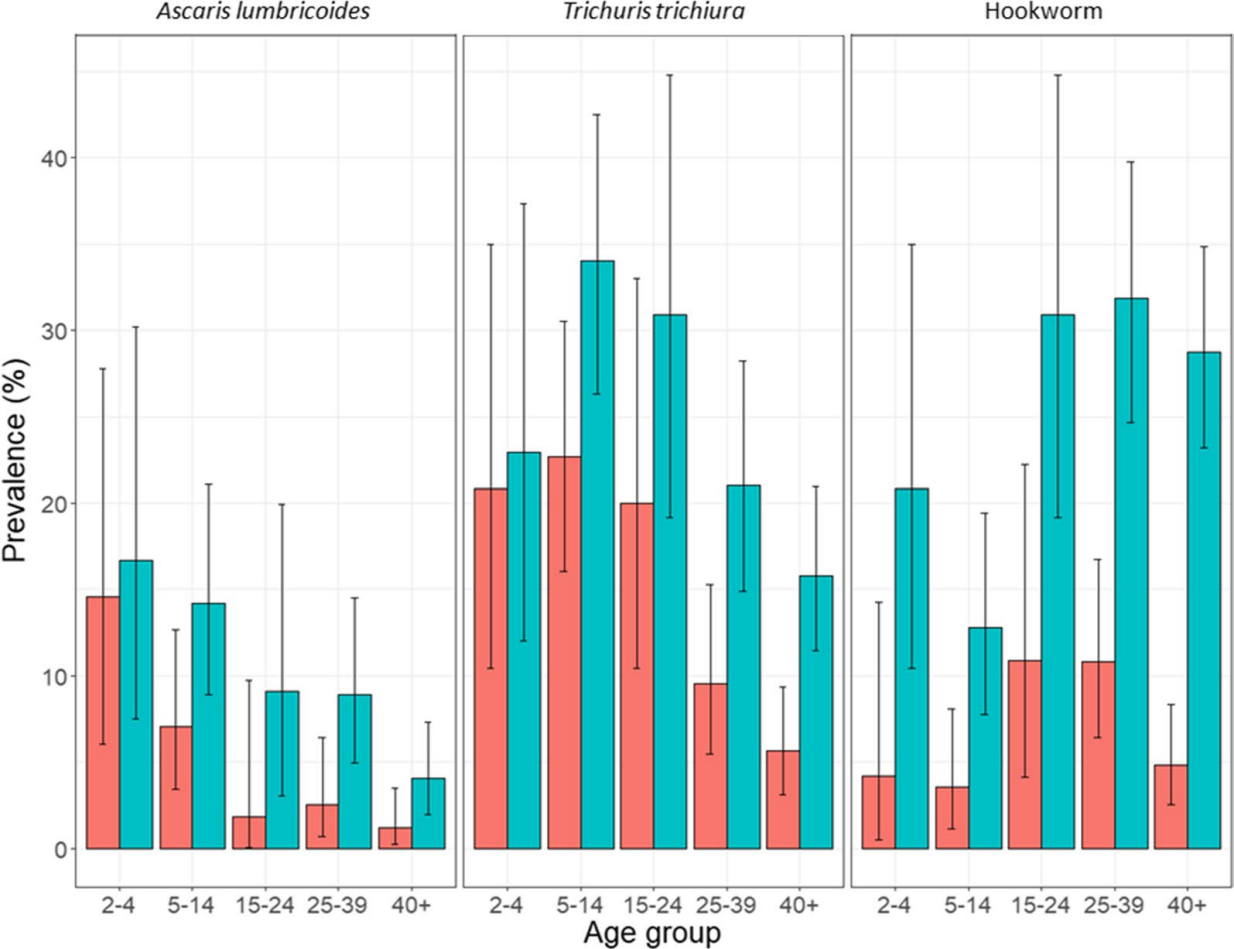
Prevalence of soil-transmitted helminths as diagnosed by the Kato-Katz technique (red) and qPCR (blue) over all age groups. Hookworm prevalence for qPCR are the aggregated results from *Necator americanus*, *Ancylostoma duodenale* and *Ancylostoma ceylanicum*. Vertical lines indicate 95% confidence intervals

The patterns of the age distributions were similar between the two diagnostic methods with prevalence peaks in preschool-aged children (pre-SAC, 2–4 years-old) for *A. lumbricoides*, school-aged children (SAC, 5–14 years-old) for *T. trichiura* and adults for hookworm. Figure 1 presents the age distribution of STH prevalence diagnosed by KK and qPCR. The largest differences in prevalence when stratified by age group, were in hookworm with a 400% increase in prevalence in pre-SAC (4.17% by KK, 20.83% by qPCR) and a 491% increase in the 40+ age group (4.89% by KK, 28.74% by qPCR).

Whilst prevalence was higher by qPCR for each species when stratified by age group, the differences were not statistically significant except for *T. trichiura* and hookworm infection in the 25–39 and 40+ years age groups, and hookworm infection in the 5–14 and 15–24 years age groups.

**Fig. 2:**
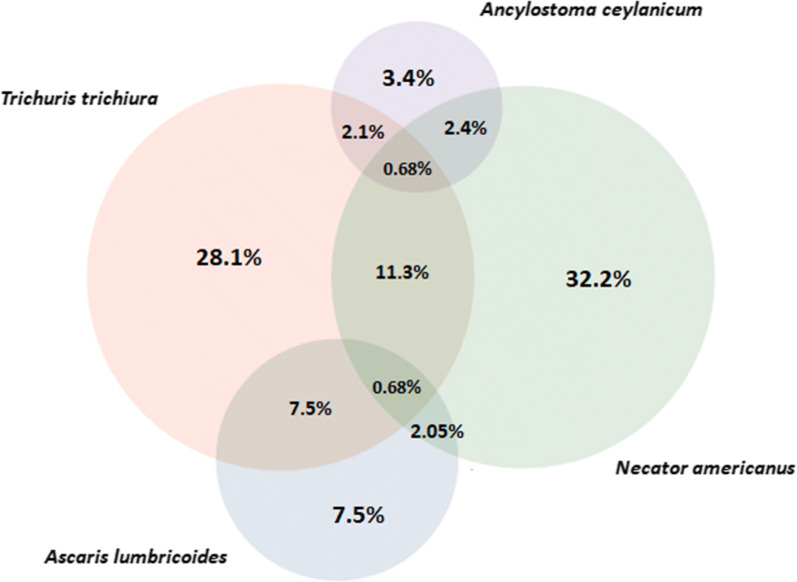
Euler diagram of soil-transmitted helminth infections and coinfections as diagnosed by qPCR (*n *= 292). Excluded from the diagram is one participant with a coinfection of *Ancylostoma duodenale* and *Necator americanus* (0.4%)

Figure 2 presents the overlap of STH coinfections as diagnosed by qPCR. Of the 292 positive infections 208 (71.23%) were single infections, 77 (26.37%) were infections with two species and seven (2.40%) with three species. Of the 84 coinfections, 44.05% (37/84) were between *T. trichiura* and *N. americanus*.

### Intensity of infection

**Fig. 3:**
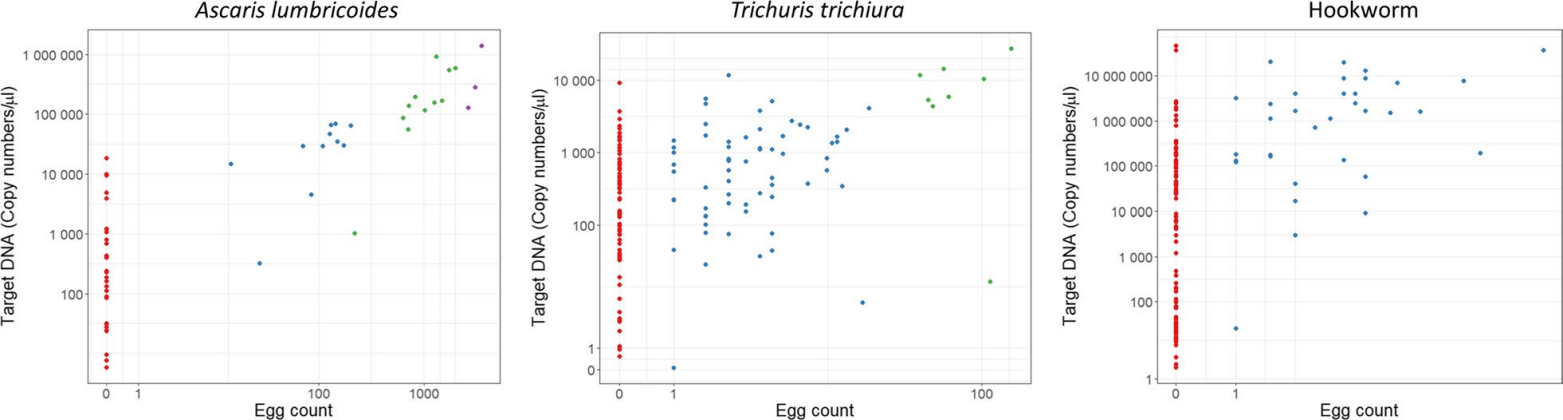
Scatterplot of *Ascaris lumbricoides* (left), *Trichuris trichiura* (centre) and hookworm (right) eggs per gram (EPG) of faeces results against mean DNA quantity results on log axes. EPG results were derived from the Kato-Katz (KK) technique. Mean DNA quantity results were derived from qPCR. Point colour denotes egg count infection intensity grouping as defined by the WHO [37]. *Key*: red, negative; blue, low; green, medium; purple, high

Mean target DNA copy numbers were calculated using the standard curve calculated during qPCR. There was evidence for a linear relationship between egg count and DNA quantity. Figure 3 shows the relationship between EPG values derived from Kato-Katz and DNA copy numbers for *A. lumbricoides* and *T. trichiura*. The correlation coefficients for the linear regression between DNA copy number and egg count were 0.81 for *A. lumbricoides*, 0.73 for *T. trichiura* and 0.48 for hookworm. This indicates a strong positive correlation for *A. lumbricoides* and *T. trichiura*. The Kendall Tau-b value was 0.68 for *A. lumbricoides*, indicating strong concordance between DNA quantity and EPG, 0.56 for *T. trichiura*, indicating moderate concordance, and 0.50 for hookworm, indicating moderate concordance. Points that lie on the x- and y-axes indicate that the two diagnostics do not agree on whether the sample was infected or not. Of the 623 KK-negative *A. lumbricoides* results, 32 (5.14%) were deemed positive by qPCR. This increased to 78 samples out of 566 (13.78%) for *T. trichiura* and 42 out of 606 (20.46%) for hookworm.

The median copy number for samples that were positive by both KK and qPCR was 68870.38 for *A. lumbricoides*, 560.46 for *T. trichiura* and 1633045.0 for hookworm. Of the samples that were negative by KK but positive by qPCR, 100% (30/30) were below the median for *A. lumbricoides*, 65.75% (48/73) for *T. trichiura* and 92.73% (102/110) for hookworm. Whilst EPG infection intensities cannot be directly calculated from the target DNA copy numbers, these results indicate that the infections missed by KK were predominately in the lower sector of the distribution of intensity of infection scores in the sample. Figure 3 also shows how the samples are grouped according to the WHO’s intensity of infection stratifications of low, medium and high [37]. In conjunction, Table 2 shows the number of infections, the mean DNA copy number per µl and 95% confidence intervals in each intensity group. The mean DNA copy number increased for each intensity group in each species (i.e. the mean for the medium intensity group is higher than the mean for the low intensity group). For *A. lumbricoides*, the mean DNA copy number within each intensity group was significantly different at *P *< 0.05 to the one that follows it except for the medium and high intensity groups (*P *= 0.496). For *T. trichiura* the mean DNA copy numbers were significantly different between the groups.

**Table 2:** Number of positive samples by qPCR (n), mean DNA copy number per µl and 95% confidence intervals, stratified by soil-transmitted helminth intensity of infection grouping

Species	Intensity group by Kato-Katz							
	Negative		Low		Medium		High	
	*n*	Mean DNA copy number/µl (95% CI)	*n*	Mean DNA copy number/µl (95% CI)	*n*	Mean DNA copy number/µl (95% CI)	*n*	Mean DNA copy number/µl (95% CI)
*Ascaris lumbricoides*	623	203.77 (83.09–423.08)	11	70598.89 (42892.01–97456.66)	11	539384.33 (303364.28–994523.32)	3	1202662.65 (256585.80–2048046.53)
*Trichuris trichiura*	566	170.28 (112.25–305.51)	74	2159.36 (1548.19–3344.78)	8	20110.37 (11082.89–35039.10)	0	.
Hookworms	606		42		0		0	
*Necator americanus*		326466.03 (55039.74–1112959.65)		7232805.61 (4158351.54–13601987.63)		.		.
*Ancylostoma duodenale*				.		.		.
*Ancylostoma ceylanicum*		18547.90 (5566.54–53187.54)		1097750.25 (292621.84–2836328.32)		.		.

*Note*: Intensity group cut-offs as defined by the WHO [37]

**Fig. 4:**
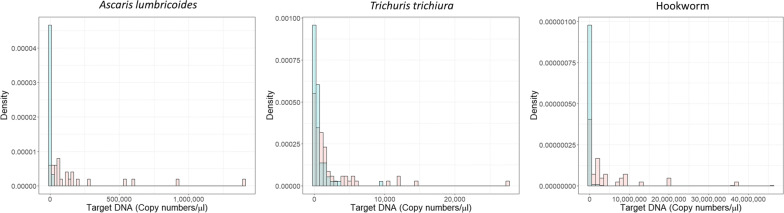
Density histogram of *Ascaris lumbricoides*, *Trichuris trichiura* and hookworm target DNA copy numbers per µl for all samples that were negative by KK (blue bars) and positive by KK (red bars)

The DNA copy numbers of all samples that were KK-negative (blue) and KK-positive (red) were plotted in a histogram to analyse the distribution (Figure 4). If the distributions from several epidemiological studies employing the qPCR diagnostic align to create a body of evidence on the probability association between the two test methods, a distribution could be applied to KK-negative results both to estimate how many would be deemed positive if they underwent qPCR and to give an estimate of DNA copy number. The distribution of copy number followed a negative binomial distribution, similar to EPG. This was especially clear for *T. trichiura* where the prevalence of infection was higher.

### Diagnostic sensitivity and methodology testing

Combining Kato-Katz and qPCR results to give a “true positive”, and assuming that specificity for both diagnostics is 100%, the sensitivity of KK and qPCR to detect *A. lumbricoides* was 45.45% and 100% respectively and, for *T. trichiura*, 52.30% and 91.61% respectively. For the aggregated hookworm results sensitivity was 25.30% for KK and 100% for qPCR.

Analysis of technical and extraction replicates showed that qPCR results were consistent across duplicates. However, it is important to note the small sample size of positive results in this section. The mean standard deviation in C_t_ values between the two wells, excluding *An. duodenale* as there was only one positive result, was 0.38. From this we can conclude that the PCR plate replicates were quantitatively consistent. To assess consistency between DNA extractions, 24 stool samples underwent duplicate extractions. All duplicate extractions were in agreement for whether the sample was infected or not infected except for one sample which tested negative for *A. ceylanicum* in extraction 1 and positive in extraction 2. The mean difference in C_t_ for all duplicates was 1.31 cycles, ranging from 0.70 for *As. lumbricoides* to 2.98 for *An. ceylanicum*. There was no statistically significant difference between mean DNA copy number for extraction 1 and extraction 2 (Table 3).

**Table 3:** Number of positive samples by Kato-Katz and qPCR, mean DNA copy number per µl and mean difference in DNA copy number per µl from samples that underwent duplicate DNA extractions

Species		Extraction 1		Extraction 2		Mean difference in DC/µl^a^	*P*-value^b^
	*n* positive KK	*n* positive PCR	Mean DC/µl	*n* positive PCR	Mean DC/µl		
*Ascaris lumbricoides*	1	3	18710.68	3	31092.67	12387.62	1.00
*Trichuris trichiura*	2	3	4359.60	3	6099.49	1739.89	0.25
Hookworms	3						
*Necator americanus*		4	46875.50	4	392866.11	346299.27	1.00
*Ancylostoma duodenale*		0	.	0	.	.	.
*Ancylostoma ceylanicum*		2	4630511.00	3	2894048.15	393997.91	1.00

^a^ Mean of the differences between each paired sample. ^b^ P-value from the significance test determining the difference between the mean DNA copy number for extraction 1 and the mean DNA copy number for extraction 2

*Abbreviation*: DC, DNA copy number; KK, Kato-Katz

## Discussion

The limitations of the Kato-Katz techniques have been well documented in the scientific literature. However, it remains the recommended and most widely used diagnostic technique because of simplicity of use and cost. The low cost of performing the test and the low demands on equipment and expertise to carry it out imply it is a very useful diagnostic in resource-poor settings. However, one of the main reasons why KK remains the STH diagnostic of choice is the lack of a better alternative. Whilst there are other microscopy techniques, such as the McMaster method and FLOTAC, these techniques are limited by the same issues as KK such as lower sensitivity in low intensity settings and a high impact of human error [29, 36]. Quantitative PCR is widely used in the biological sciences (and in infectious disease epidemiological studies involving viral, bacterial and protozoan infections) to detect target DNA and quantify it in a sample. The neglected tropical diseases (NTDs) have lagged behind other infectious disease disciplines in applying PCR as a diagnostic tool in epidemiological studies. In this study, we have used qPCR for STH diagnosis, building on the work of researchers such as Easton et al. [16], Pilotte et al. [21] and Papaiakovou et al. [32, 37], to explore its effectiveness in a low prevalence and low intensity setting, and to further elucidate the STH infection profile of the two study villages in Myanmar.

KK and qPCR results from 648 study participants were compared to assess how the measurement of prevalence changed when using different diagnostic techniques. Prevalence of any STH by KK was 20.68% and by qPCR was 45.06%. Prevalence of any STH in SAC by qPCR was 46.81%, compared to 29.08% by KK. Whilst this is a substantial increase, it does not affect the frequency of MDA that should be applied in the Myanmar setting as recommended by the WHO based on prevalence groupings. MDA should continue at its current frequency (twice a year) [35]. Studies in Kenya [16], Timor-Leste [25], Ecuador [20] and Bangladesh [38] have all found qPCR to have greater sensitivity for detecting STH infections than KK. Unfortunately, due to time and expertise constraints, in this study we were only carrying out one KK slide per sample instead of the recommended two. A meta-analysis by Nikolay et al. [29] found that sensitivity of 1-slide KK for hookworm, for example, was 59.5% compared to 63.0% for 2-slide KK. The one sample KK approach will therefore have an impact on the comparison to the qPCR diagnostic. However, the sensitivity of qPCR is not assumed to always be 100%. In this study the sensitivity of qPCR for *T. trichiura* was 91.61%. In the Easton et al. study, the investigators compared the number of expelled worms to ng DNA/µl and found multiple instances of individuals found to be positive by qPCR who did not expel any worms. Admittedly, worm expulsion is difficult to complete due to participant study fatigue and the long timespan (many days) in which worms can be expelled after treatment. However, these results may raise questions about the “gold-standard” validity of qPCR if there is the possibility of false positive results or of qPCR accurately detecting STH DNA but in participants that do not have an active infection [39–42]. The more likely explanation of the results is that all worms had not been expelled by treatment combined with too short a period of stool collection post-treatment.

The greatest magnitude of change in the comparison of the two diagnostics was for hookworm, where prevalence increased by 295.37%. In this analysis, the sensitivity of KK was lowest for hookworm, at 25.30%. A study by Easton et al. [16], found a similar sensitivity level (for *N. americanus* only) at 28%. The study of Easton et al. also reported an increase in prevalence of 154.17% for hookworm. KK therefore appears to be the least sensitive for hookworm infections compared to other STH species. After preparation, which includes stool sieving and staining, slides should be read within half an hour for hookworm before the eggs are destroyed and are no longer visible under the microscope. In this study, the prevalence of *An. ceylanicum* was 4.63%, making up 16.85% of the total hookworm infections. *Ancylostoma ceylanicum* has previously been classified as a nematode that infects dogs and cats [43]. However, there is increasing evidence that *An. ceylanicum* is an important pathogen of humans as well. A study by O’Connell et al. on Myanmar refugees living in Thailand found a baseline prevalence of 5.4% for *An. ceylanicum* [44]. Hookworm species cannot be differentiated under the microscope, so it is likely that many studies have been including *An. ceylanicum* in hookworm prevalence values whilst using microscopy techniques. The presence of *An. ceylanicum* in the study sample could indicate transmission of infection occurring between humans and dogs and/or cats. Studies on animal stool samples from the same study sites would be required to elucidate this further. Hookworm infections in this study were mostly comprised of *N. americanus* and *An. ceylanicum*, with minimal *An. duodenale* infections. This pattern is in agreement with another molecular study in Myanmar, conducted in 2015 [45].

Morbidity from STH infection is linked to the worm burden in the human host. Higher intensity infections (greater number of worms) will result in greater morbidity due to the compounding of effects and the length of time that a person has been infected to accrue such a high burden. Therefore, quantifying STH intensity of infection in study populations is useful to determine the risk of serious morbidity. KK uses a proxy measure for STH infection by counting the number of eggs in a set amount of stool and scaling that up to give eggs per gram of faeces (EPG). If worm expulsion studies have been performed the average egg output can be converted into a worm burden estimate. Quantifying infection by PCR is more challenging and less straightforward.

PCR detects targeted DNA sequences in a sample. In quantitative PCR, a standard curve is built from positive controls that have a known number of copies of the target DNA, serially diluted to give different concentrations. The C_t_ values, and therefore the copy numbers present, of the unknown sample is compared to the C_t_ values on the standard curve to estimate the number of target DNA copies present in the sample through linear regression. There was evidence for a linear relationship between DNA copy number and EPG for *A. lumbricoides* (Kendall Tau-b = 0.68) and weaker evidence for *T. trichiura* (Kendall Tau-b = 0.56) and hookworm (Kendall Tau-b = 0.50). This magnitude of association is lower than found by Easton et al. in Kenya (Spearman’s rank correlation coefficients between DNA (ng/µl) and EPG of 0.83 for *A. lumbricoides* and 0.55 for *T. trichiura*) but higher than reported by Benjamin-Chung et al. in Bangladesh (Tau-b value between qPCR C_t_ and EPG was − 0.44 for *A. lumbricoides* and − 0.25 for *T. trichiura*) [38]. It is important that there is evidence for association between the two intensity measures, as it infers that the higher amount of DNA released by a higher number of eggs is being detected by qPCR. However, taking possible measurement error into account, it is still not recommended to estimate an EPG value from the results of qPCR and thereby estimate the worm burden and risk of morbidity from qPCR results. More comparative studies are needed before this step is taken.

In this analysis, we compared DNA copy number per µl of each sample to its matched EPG intensity group (as defined by the WHO) which found an increasing, and significantly different, mean DNA copy number with each increasing intensity group. This suggests that qPCR results can be used to group infections into low, medium and high intensity. Similar results were found by Benjamin-Chung et al. [38] using C_t_ values instead of DNA copy numbers; the caveats this entails have been previously reported.

Whilst qPCR is more sensitive than KK for detecting STH infections, there are several technical limitations that may prevent it from being classed as a “gold-standard”. In this study there was no significant difference between mean DNA copy number for duplicate extractions, and the mean difference in C_t_ values between duplicate wells was minimal. This is encouraging and suggests that when the DNA extraction and qPCR protocols are done correctly and systematically, then minimal variation is introduced. As discussed above, there remains the possibility of false-positives, supported by evidence from the Easton et al. [16] worm expulsion study. Surprisingly, the study in Bangladesh by Benjamin-Chung et al. [38] found that the detection rate for *A. lumbricoides* was 14% lower by qPCR than KK. Although the authors equate this finding to misclassification of *Ascaris* ova during microscopy, the possibility of false positive results by qPCR was not ruled out [38]. This leads on to the question of whether qPCR is always detecting active infections. An opinion piece by Papaiakovou et al. [24] discusses the questions posed by qPCR results that detect DNA copy numbers below the threshold of those from a single egg. It is possible that the DNA detected does not come from an egg but may be from other “free” DNA in the sample [40, 42]. This could be contamination from the environment or DNA from a piece of the worm in question.

Whilst the reasons listed above are important to consider when interpreting qPCR results, the growing body of evidence suggests that qPCR is much more sensitive than KK at low prevalence and intensities of infection and therefore a more reliable diagnostic tool. Nevertheless, limitations of qPCR do not stop at technical issues. One of the main barriers for using qPCR as a main diagnostic is the cost and the work hours required per sample. Whilst more low-income countries are building and staffing molecular diagnostic laboratories, reducing start-up costs on laboratory equipment, PCR consumable materials (such as primers, probes and DNA extraction kits) are costly and often difficult to import into countries in regions of endemic infection. Whilst several studies have managed to decrease the cost per sample of qPCR to around the same price as KK, the laboratory work for these studies was completed with the help of centres in high-income countries and does not include the cost of labour [20, 46].

In conclusion, the increasing body of evidence that qPCR is significantly more sensitive than KK must be considered when designing future STH monitoring and evaluation activities, particularly as many countries move to a low prevalence state after many years of MDA. Currently the cost of qPCR is a significant barrier to its widespread application. With a concerted effort by research institutions and ministries of health, the bulk costs of equipment and consumables could be brought down, allowing low-income countries to apply qPCR as a validation tool for surveillance based on KK results. Modelling studies have shown that interruption of transmission is likely when areas reach a true prevalence threshold of 2%. If we are to design control programmes that include this threshold, qPCR is essential to accurately and reliably prove that countries are reaching the transmission interruption threshold and, in the future, certify that elimination has been achieved.

## Conclusions

This study highlights the major differences between prevalence levels when diagnosed by KK or by qPCR especially in low intensity infections. The data presented here also provides evidence of a linear relationship between EPG and DNA copy number, as well as associations between intensity groupings based on KK and DNA copy number. The information here adds to the literature on STH diagnostics, an area much in need of attention, and opens interesting avenues for the selective application of qPCR in STH M&E activities as the impact of widespread MDA in endemic regions increases over the coming decade.

## Data Availability

The datasets used and analysed during the current study are available from the corresponding author on reasonable request and pending approval from all stakeholders.

## Abbreviations

BCa: bias-corrected and accelerated
CI: confidence interval
C_t_: cycle threshold
DMR: Department of Medical Research, Yangon, Myanmar
EPG: eggs per gram of faeces
GSK: GlaxoSmithKline
ICREC: Imperial College Research Ethics Committee
ID: identification
KK: Kato-Katz
LCNTDR: London Centre for Neglected Tropical Disease Research
MDA: mass drug administration
M&E: monitoring and evaluation
NTD: neglected tropical disease
PCR: polymerase chain reaction
PPV: positive predictive value
POC-CCA: point of care-circulating cathodic antigen
Pre-SAC: preschool-aged children
QC: quality control
qPCR: quantitative polymerase chain reaction
SAC: school-aged children
STH: soil-transmitted helminths
UoPH: University of Public Health, Yangon, Myanmar
WHO: World Health Organization
